# Monolithic Columns for the Isolation of Lectin: Principles, Advances and Prospects

**DOI:** 10.1021/acsomega.5c11664

**Published:** 2026-02-03

**Authors:** Ivonéa Soares do Nascimento, Charline Soares dos Santos Rolim, Ana Cristina Freitas de Oliveira Meira, Jaime Vilela de Resende, Renata Cristina Ferreira Bonomo, Cristiane Martins Veloso, Rafael da Costa Ilhéu Fontan

**Affiliations:** † Process Engineering Laboratory, Universidade Estadual do Sudoeste da Bahia, Itapetinga 45700-000, Brazil; ‡ Food Refrigeration Laboratory, Department of Food Science, Universidade Federal de Lavras, Lavras 37200-900, Brazil

## Abstract

Cryogels are materials formed from the polymerization of monomers under freezing conditions and are mainly used as monolithic chromatography columns. Depending on the functionalization technique, they can be used for ion exchange chromatography, immobilized metal ion affinity chromatography (IMAC), hydrophobic interaction and more selective affinity methods. Due to their physical, chemical and hydrodynamic properties, cryogels find applications in various fields, such as the food industry for purification processes, the pharmaceutical, medical/biomedical and environmental industries, for the removal of waste or toxic substances from the environment. Affinity chromatography is a widely used method in liquid chromatography, and cryogels are closely related to it. Therefore, the aim here is to describe and analyze advances in the development of supermacroporous monolithic columns and their main applications focused on affinity chromatography. Thus, there is a vast field involving monolithic columns for application in affinity chromatography aimed at isolating biomolecules, including lectins. Lectins are glycoproteins with the ability to bind to carbohydrates and perform various functions such as antibacterial, antitumor, immunomodulatory and antiviral responses, among other applications. This review allows us to emphasize the great advances in the development and application of cryogels, materials that have wide applicability in various areas, such as food, biological, medical, biomedical, pharmaceutical and environmental. It is a product that is easy to synthesize and reproduce.

## Graphical abstract

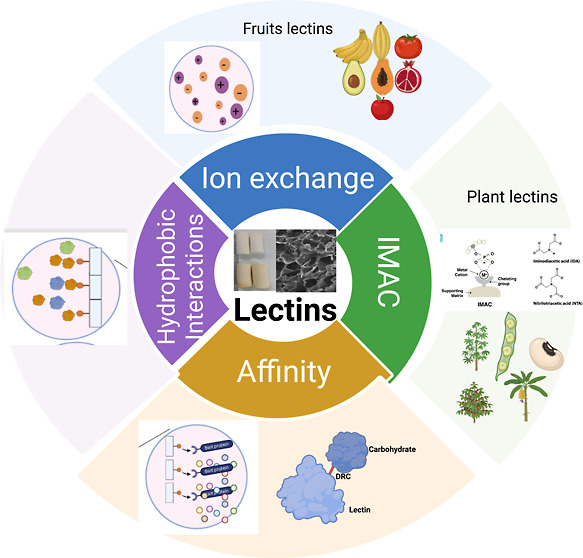

## 1 Introduction

Technological advances in the pharmaceutical and food industries have brought gains to the population. Increasingly, the use of active biocompounds that maintain their characteristics is being sought. To this end, some techniques are used, such as chromatography, which is commonly used to separate biomolecules of interest from other compounds present in a given sample under study.^1,2^

According to Figure 1, the purification of biomolecules by chromatography can be carried out through separation based on different principles, such as ion exchange, hydrophobic interaction or affinity. The choice of one or more of these techniques depends very much on the molecule of interest. As pointed out by Machado et al.,^3^ the fixed bed technique is recognized for its high efficiency and ease of implementation in production processes.

**Figure 1:**
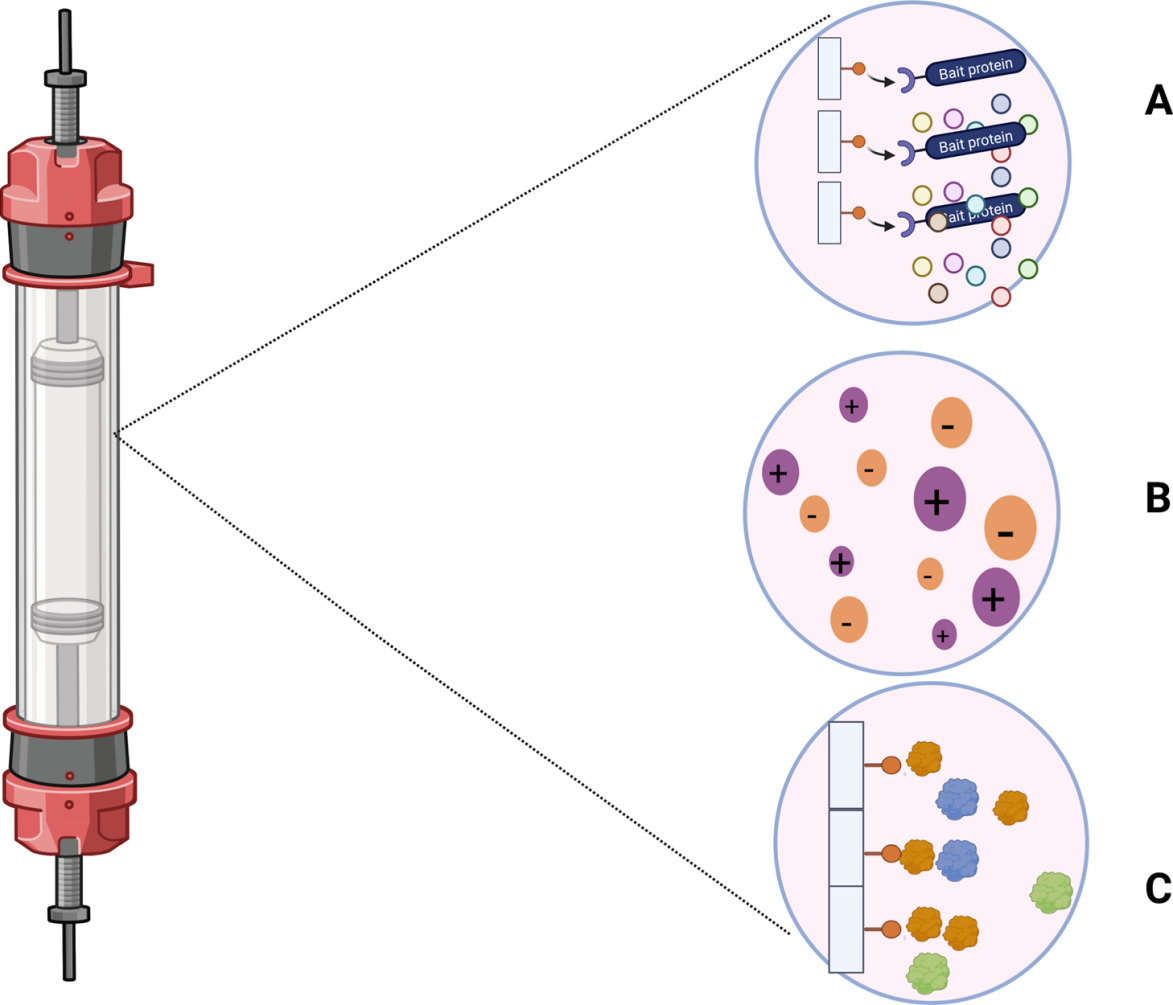
Chromatography Methods in the Purification of Biomolecules. Representation of a liquid chromatography column, an essential method for separating mixtures. The three magnification windows detail the main separation mechanisms that can occur within the column, based on the interactions between the sample (mobile phase) and the filling material (stationary phase). (A) Affinity chromatography, where there is a specific interaction between the mobile and stationary phases. (B) Ion Exchange Chromatography, in which the interaction occurs due to differences in the electrical charges of the molecules. (C) Hydrophobic Interaction Chromatography, in which the interactions between the stationary phase and mobile phase occur due to hydrophobicity, i.e., the aversion of molecules to water.

Thus, studies have been carried out to solve the problem involved in fixed beds, focusing on the development of new materials to meet the need for a material that can be used to purify large molecules or even highly concentrated materials in significant quantities. In this context, the so-called cryogels have emerged, which are supermacroporous monolithic columns synthesized from a polymerization reaction under freezing conditions.^2,4^

Polymeric monolithic columns refer to supports that have structures without a single body, with a wide and interconnected network of pores, and are used for the separation and purification of bioproducts from unclear media or solutions containing particles or mixtures of cells. Cryogels have multiple pores, with dimensions that can vary between 1 and 100 μm in diameter. These dimensional characteristics can be controlled by modifying the synthesis parameters, which include the nature and type of the polymer, the synthesis temperature, and the composition of the cross-linking agent.^4,5^

Affinity chromatography is based on the interaction of the molecule of interest with a specific ligand fixed to the column, providing greater specificity and selectivity in chromatographic processes. During the synthesis of the cryogel, this ligand is incorporated into the matrix at the time of functionalization.^4^ In this context, it is possible to introduce a type of ligand of interest, such as the addition of a sugar (*N*-Acetyl-d-glucosamine) for the purification of lectins.^6^ Based on these principles, this study aims to describe and analyze advances in the development of supermacroporous monolithic columns and their main applications in affinity chromatography for the isolation of lectins.

## 2 Supermacroporous Monolithic Columns—Cryogels

Cryogel is a type of matrix commonly used in chromatography for the separation and purification of biomolecules, characterized by an interconnected porous structure that forms a single body. As with the chromatography technique, there are various columns developed through the cryogelification process, which can be ionic, affinity or hydrophobic in nature^2,4,7^

According to Jones et al.,^8^ cryogel synthesis is based on the polymerization reaction of monomers in a frozen environment. These can be produced from any gel-forming precursor and with a wide variety of morphologies and porosities as shown in Figure 2.

**Figure 2:**
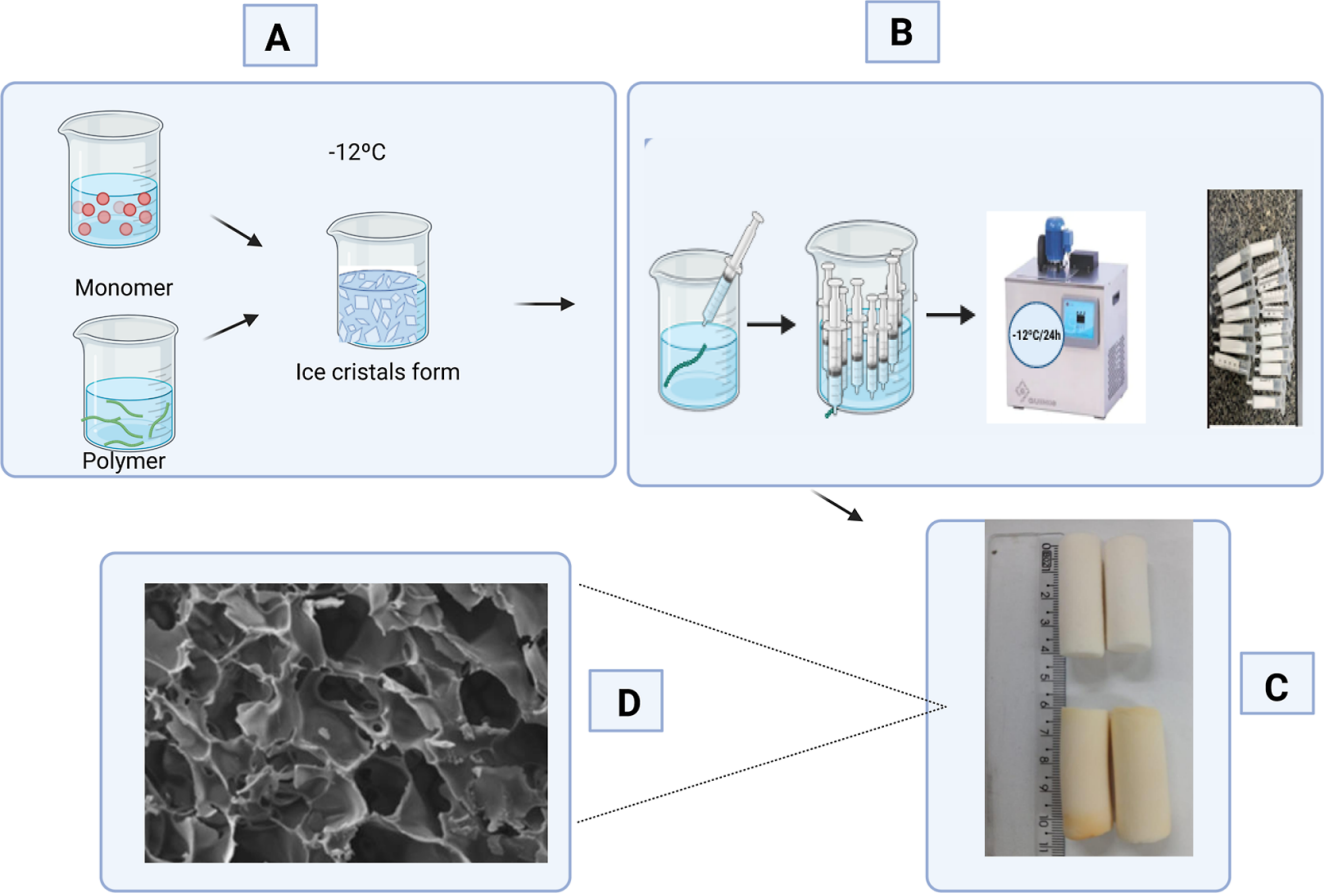
Cryogel synthesis. The figure represents the basic synthesis of cryogels made from acrylamide (Aam), bis-acrylamide (BAam), allyl-glycidyl ether (AGE), *N*,*N*,*N*,*N*-tetramethylethylenediamine (TEMED) and ammonium persulfate (APS). (A) mixture of monomers responsible for the polymerization reaction, which occurs at low temperatures of −12 °C. (B) Packaging of the solution in 10 mL syringes and polymerization reaction occurring in a bath at −12 °C for 24 h. (C) Shapes of cryogels produced after polymerization, thawing and drying in an oven, which can vary from 4 to 5 cm in length with a diameter of 1.4 cm. (D) Microscopic structures of cryogels showing their porosity, which varies in size and distribution of pores, a characteristic feature of cryogels.

For synthesis processes, many monomers are used to obtain the cryogel matrix, as long as they have the ability to form gels. A polymer widely used in this case is polyacrylamide, which is the result of the polymerization of two gel-forming monomers, Bis-acrylamide (BAam) and Acrylamide (Aam). In some cases, another monomer, allyl-glycidyl ether (AGE), can be added to provide epoxy groups useful for the functionalization process to graft chemical groups that will determine the chromatographic method to be used, be it ion exchange, hydrophobic, affinity or other.^2,9,10^

The structure of the cryogel depends very much on the monomers used, their concentration and the temperature used in the cryofreezing process. The variability of these factors influences the quantity, distribution and pore sizes of the matrix. Acrylamide allows the chain to be linear, while bis-acrylamide has the cross-linking capacity of the chains generated by acrylamide, resulting in the cross-links that make the gel hold together. When AGE is used, it increases the strength of the gel and makes reactive epoxy groups available on the surface of the monolithic matrix, which can then be used for functionalization processes.^6,7^

There are studies in the literature that study different concentrations and types of monomers used in the synthesis of cryogels, as well as variations in the temperature and pH of the solution. The variation in temperature influences the process of ice crystal formation and consequently the size and variation of the pores.^11^ High temperatures generate faster crystallization with smaller and smoother pores and at temperatures of −20 °C and −80 °C the pore size went from 75 to 58 μm, while at −5 °C, −10 °C and −22 °C there was a reduction from 55 to 23 μm.^12^

The concentration of the monomers used in the synthesis of cryogels is of fundamental importance when assessing the porosity of the matrix. Increasing the concentration of the polymer reduces the porogenic agent in the medium, in this case water, which under freezing and then thawing conditions leads to the formation of pores. Just as a decrease in the concentration of the polymer increases the availability of water in the medium and consequently increases the size and availability of the pores.^11^

Ferreira da Silva et al.^13^ sought to optimize the immobilization of sugars on the surface of cryogels using the glutaraldehyde method, varying the concentrations of acrylamide, bis-acrylamide and AGE and the influence of temperature during the synthesis process. Thus, it was observed that the temperature of the glutaraldehyde method and the concentration of the carbohydrates influenced the amount of sugar immobilized in the column. Some reagents such as *N*,*N*,*N*,*N*-tetramethylethylenediamine (TEMED) and ammonium persulfate (APS) are used in cryogel synthesis to initiate and accelerate the polymerization reaction. When placed in the presence of water, ammonium persulfate promotes the formation of free radicals, which, when placed with acrylamide, promote a radical reaction.^6,7,14^

There is currently a range of monomers that are used in cryogel synthesis, the choice of these materials depending very much on the biomolecule of interest to be obtained through purification. Chen et al.^15^ used Lauryl methacrylate (LMA) as the monomer and Divinylbenzene (DVB) as the cross-linker, a mixture of Benzoyl peroxide (BPO) and *N*,*N*-Dimethyl aniline (DMA) as the reaction initiators. Other studies have used a range of monomers such as 2-hydroxyethyl methacrylate (HEMA), *N*,*N*-methylene-bis­(acrylamide) (MBAA),^16^ 2-hydroxyethyl methacrylate (HEMA), glycidyl methacrylate (GMA) and MBAA.^17^ Figure 3 shows some of the polymers used in cryogel synthesis.

**Figure 3:**
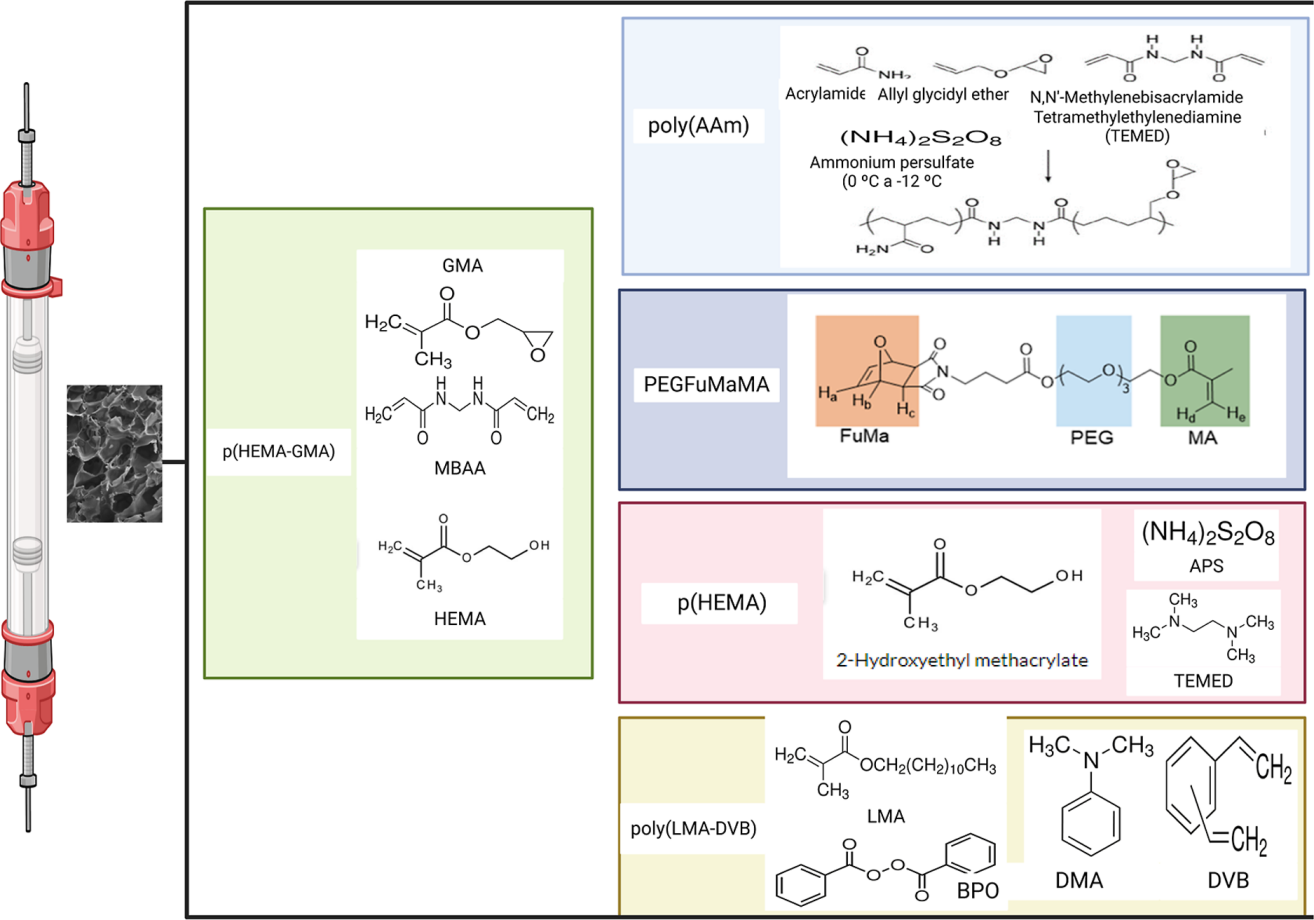
Polymers used in cryogel synthesis.

## 3 Surface Modification of Cryogels

Cryogels have a smaller surface area when compared to commercial chromatographic columns, which is a negative point for chromatographic columns, so many studies are being carried out to improve the problematic size of the surface area of cryogels, for this, methods called functionalization that are carried out by chemical or physical means are being used to increase the surface area of cryogels.^2–4,6,10^

Cryogel functionalization techniques include activation of the monolithic column by ionic interactions, immobilization via covalent bonding, biospecific adsorption, among others.^4,18,19^ For each type of reactive group that is to be functionalized on the surface of a column, the main point is the characteristic of the biomolecule that is to be separated by the chromatography process, so, as can be seen in Table 1, there are many techniques and ligands used in the most diverse studies to date 4. In general, the methods commonly used for affinity chromatography are the immobilization of ligands via covalent bonding. Of these, three are the most widely used in the literature: the epoxy method, the Schiff base method and the glutaraldehyde method.^2,6,9,20,21^

**Table 1:** Cryogel Functionalization Techniques^a^

technique	binders	application	references
glutaraldehyde method	β-d-galactosidase immobilized on chitosan	continuous hydrolysis of lactose and the synthesis of galactooligosaccharides (GOS)	Klein et al.^26^
covalent bond	iminodiacetic acid (IDA)	bromelain purification	Porfirio et al.^25^
	anion exchange ligands [2-(dimethylamino)ethyl group]	purification of Escherichia coli Cells	Arvidsson et al.^27^
	Cu^2+^ binding	purification of cytochrome *c*	Çimen and Denizli^20^
	P-Tyr amino acid	purification of IgG from human serum	Mourão et al.^21^
enzyme immobilization, epoxy method	human serum albumin (HSA)	purification of biomolecules	Mallik, Jiang and Hages^22^
chemical coupling	α-chymotrypsin	Enantioselective hydrolysis of a Schiff base of d,l-phe-OEt (D,L-SBPH)	Belokon et al.^28^
epoxy method	immobilized polyethyleneimine (PEI)	capture of bacterial endotoxins (BEs)	Hanora et al.^29^
	polymyxin B (PMB) and lysozyme		
grafting of ionic groups	cationic (AMPSA and AAc) or anionic (AETA-Q and DMAEMA) exchangers	jackfruit lectin purification	Nascimento et al.^2^
schiff base	IDA Cu^2+^ binding	capture of lactoferrin from cheese whey	Carvalho et al.^9^

a Source: Adapted from Silva et al.^4^

When AGE is used in its synthesis, cryogels have epoxy radicals in their structure. In this case, this radical undergoes a nucleophilic attack, resulting in the formation of a secondary amine due to this interaction. Subsequently, these radicals are inactivated by means of a compound containing an amine group, in order to avoid undesirable bonds during this process. Usually, the compound used in this method is ethanolamine.^6,22^

Functionalization using the Schiff Base method uses the epoxy radical present in the cryogel, which is converted into diols. In this way, the epoxy ring is opened and converted into diole groups, which are then oxidized, resulting in aldehydes. This type of reaction can take place using periodic acid, for example. The aldehydes resulting from this reaction bind to the amine groups of the ligands, resulting in a Schiff base. This base is used in functionalization processes, being converted into a stable secondary amine by reduction, commonly with sodium cyanoborohydride or sodium borohydride.^23,24^

In the glutaraldehyde method, the active epoxy radical provided by allyl glycidyl ether (AGE) is transformed into an activated amine under the influence of substances containing amine groups in their structure, such as ethylenediamine. The latter, in turn, reacts with glutaraldehyde, becoming an activated aldehyde that can then react with any component of interest that contains amine groups in its structure.^2,6,23^

Figure 4 shows the copolymerization reaction that occurs in the synthesis of cryogels and the glutaraldehyde method used to functionalize some cryogels, especially those intended for use in affinity chromatography. This method is widely used to functionalize cryogels, because unlike other methods, at the end of this reaction, the AGE plus the glutaraldehyde allow the formation of spacer arms that allow the binding of molecules of interest without the effect of steric hindrance, thus favoring a large amount of immobilization of the ligand of interest in the cryogel matrix and consequently a higher yield in the adsorption processes.^2,6,23,25^

**Figure 4:**
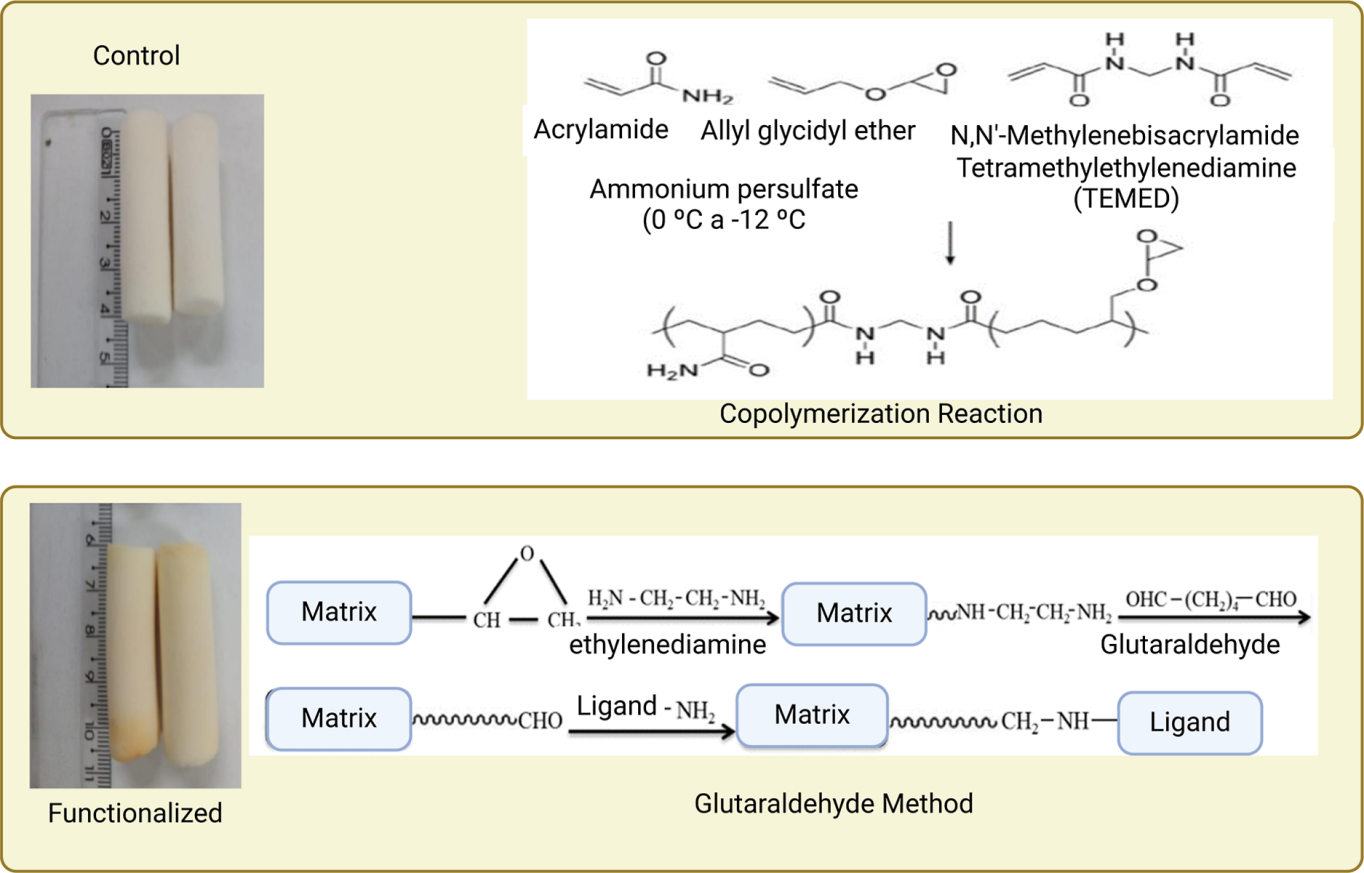
Copolymerization and functionalization reaction using the glutaraldehyde method.

## 4 Affinity Liquid Chromatography Using Cryogels

Cryogels are materials with supermacroporous spongy structures, with potential applications in various areas, due to a range of properties that these monoliths possess, such as good elasticity, high porosity, high permeability, good convective mass transfer.^27,30^

Several studies have shown the potential of using this type of monolithic column for preparative chromatography.^2,4,6,7,10,30^ Among the various techniques used in chromatography, affinity chromatography is the one that stands out the most because it is an easy technique to carry out in most laboratories.

Some of the main applications of affinity cryogels can be their ability to deliver nutrients and eliminate some metabolic residues from cell growth, due to their flexibility.^31^ Another application of monolithic columns is for use as a solid phase matrix in chromatography to isolate or remove (bio) macromolecules such as enzymes,^32^ contaminants, drug carriers, proteins, tissue engineering.^33–35^

### 4.1 Medical/Biomedical Application

Ingavle et al.^19^ sought to synthesize four different cryogel formulations (poly-PVA, poly-Am-AGE, poly-HEMA-MBA and poly-HEMA-PEGDA) and functionalize them with an antibody (protein A) to remove the anthrax toxin antigen (AP). It was possible to establish that the poly-Am-AGE cryogel has properties that favor binding to this antigen and can therefore be used in processes that favor the reduction of these antigens.

Santos et al.^36^ worked with poly-2-hydroxyethyl methacrylate (poly-HEMA) cryogels, MBAAm as a cross-linker, APS and TEMED as initiators and accelerators of the polymerization reaction. The aim of this study was to purify the sc isoform of the NTC7482-41H-VA2 HA plasmid expressing the influenza virus HA gene.

Bektas et al.^37^ synthesized cryogels based on locust bean gum (LBG), xanthan gum (XG) and mastic gum (MG), using these polymers in combination LBG-XG (LX) and LBG-XG-MG (LXM). The aim of the study was to prove the effectiveness of cryogels for use as frameworks for cartilage tissue engineering and drug release. The results proved through physical, mechanical and chemical properties that LX and LXM precursor cryogels are strong candidates for such purposes.

Ulusoy et al.^38^ purified beauvaricin from fungal extracts using a cryogel synthesized from 2-hydroxyethyl methacrylate (HEMA) and *N*,*N*,-methylenbisacrylamide (MBAA) as monomers and APS and TEMED as a precursor and accelerator of the polymerization reaction imprinted with beauvaricin (BEA). The results were promising, with an adsorptive capacity of 43 mg g^–1^ of cryogel.

In a study by Hüseynli et al.^39^ using a cryogel developed with the monomers 2-hydroxyethyl methacrylate (HEMA) and *N*,*N*-methylene bisacrylamide (MBAAm), activated with anti-HSA Fab for the purification of human serum albumin (HSA), this objective was achieved using immunoaffinity chromatography. The PHEMAC-Fab cryogel has a larger surface area and a higher macropore ratio when compared to the PHEMAC cryogel, thus favoring chromatography processes.

### 4.2 Food Industry

Much of the work involved in the food industry focuses on the use of biomolecules in various areas of this field. This is the case of the study carried out by Bereli et al.^33^ who synthesized a supermacroporous cryogel based on poly­(hydroxyethyl methacrylate) printed with l-histidine used for the purification of lysozyme from egg white, since this enzyme has various applications and in the food industry can be used as a food additive in dairy products.

Perçin et al.^40^ managed to purify the bean lectin (*Canavalia ensiformis*) Concanavalin A (Con A), using as a chromatographic column a cryogel synthesized with poly­(hydroxyethyl methacrylate) (PHEMA) functionalized with the mannose ligand. The study by Erol et al. (2019)^41^ used a cryogel with poly­(2-hydroxyethyl methacrylate-glycidyl methacrylate) (poly­(HEMA-GMA)) to evaluate the immobilization of catalase, an enzyme widely used in the food industry. The results showed that the adsorption capacity of catalase reached 298.7 ± 9.9 mg g^–1^ after 9 h of using the poly­(HEMA-GMA)-250 cryogel, so this column can be used in catalase immobilization processes so that it can be applied in the food and biological industries.

Cristina Oliveira Neves et al.^42^ used a polyacrylamide cryogel functionalized with l-tryptophan (cryogel-Trp) and l-phenylalanine (cryogel-Phe) to purify ora-pro-nobis protein (OPN) from crude leaf extract. Cryogel-Phe showed the highest adsorption capacity, reaching 92.53 mg g^–1^. Consequently, this column exhibits characteristics that make it suitable as a chromatographic support for the purification of OPN proteins derived from the crude leaf extract.

Dragan et al.^43^ developed a cryogel based on poly­(*N*,*N*-dimethylaminoethyl methacrylate), in which curcumin (CCM) was encapsulated in acrylamide networks using the cryogeleification technique. Subsequently, they functionalized the semi-IPN cryogels with monochlorotriazinol-β-cyclodextrin (MCT-β-CD), known as NPI. The results indicated that semi-IPN cryogels showed a faster release of MCC compared to IPN cryogels. This approach can be extended to encapsulate other substances and control their release in the gastrointestinal tract.

Eren et al.^44^ developed a cryogel based on 2-hydroxyethyl methacrylate, called cryogel (PHEMA-VIM), synthesized in the presence of 1-vinylimidazole as an affinity ligand for the purification of laccase produced by the fungus *Aspergillus niger*. The purification factor was calculated as 10.53 under optimal conditions, and the enzyme recovery reached 86.7% from the fermentation medium. This monolithic column appears to be a promising candidate for industrial applications, representing a less robust step.

### 4.3 Pharmaceutical Industry

Due to its porous characteristics, cryogel is often compared to a sponge-like structure, which is why some studies have sought to use this matrix to be used in the pharmaceutical industry as a drug carrier.

The study carried out by Kim et al.^45^ used an injectable cryogel in which heparin was conjugated to gelatin to carry vascular endothelial growth factor (VEGF) and fibroblasts in ischemic hind limb disease. The results were promising and the cryogel presented sponge-like characteristics and its controlled VEGF delivery capacity allowed vascular recovery and necrosis of the lower limbs.

Momekova et al.^46^ developed a cryogel using 2-hydroxyethyl cellulose (HEC) and β-cyclodextrin (β-CD) designed for the topical release of cannabidiol (CBD). The cryogels demonstrated a biphasic release pattern, characterized by a rapid initial release in the first 3 h, followed by a more gradual release of the drug. This release profile can be considered advantageous in the context of treating neoplastic skin conditions.

Ari et al.^47^ developed a poly­(β-cyclodextrin) cryogel with blood compatibility and partially hydrolytic degradability. This poly­(β-CD) material was able to gradually and simultaneously release the drugs tested, in this case hydrophilic vancomycin and hydrophobic tetracycline.

### 4.4 Removal of Environmental Contaminant Residues

The removal of environmental contaminants using cryogels has proven to be a promising approach due to their high porosity, mechanical stability and selective adsorption capacity. These polymeric materials, obtained by freezing and subsequent lyophilization, have a highly porous three-dimensional structure, allowing the efficient capture of pollutants, such as heavy metals, dyes and persistent organic compounds, in aqueous matrices. In addition, cryogels can be functionalized with specific agents to increase their affinity with certain contaminants, making them sustainable and effective alternatives for environmental decontamination processes.^4,13,48^

Tamahkar et al.^49^ used a cryogel based on poly­(hydroxyethyl methacrylate)-PHEMA synthesized in the presence of a functional monomer, *N*-methacryloyl-histidine methyl ester (MAH), for the selective removal of Ni­(II) ions from aqueous solutions. The importance of this study lies in the development of ion-imprinted supermacroporous PHEMA cryogels, which demonstrated high selectivity and efficiency in the removal of Ni­(II) from aqueous solutions. Nickel is a heavy metal widely used in industrial processes and can cause environmental impacts and harm to human health when present in contaminated water. The selectivity of cryogels in relation to other competing metal ions, such as Fe­(III), Cu­(II) and Zn­(II), indicates their potential as a sustainable technology for industrial effluent treatment. Furthermore, the reusability without significant loss in adsorption increases the economic and environmental viability of this material, making it a promising alternative for the selective removal of heavy metals in water decontamination processes.^49^

Şarkaya et al.^50^ in a study aimed at removing heavy metal ions from aqueous media used a cryogel based on poly­(hydroxyethyl methacrylate)-PHEMA with silver ions (Ag) using *N*-methacryloyl-l-cysteine as a functional monomer. In this case, it was possible to obtain an adsorption of 49.27 mg g^–1^, in addition, it was possible to evaluate the reusability of this column, since the adsorption test was performed for more than 10 consecutive cycles without any loss or decrease in the adsorptive capacity of the monolith.

The study conducted by Evli et al.^51^ developed a cryogel based on poly­(acrylamide-*co*-methylmethacrylate) functionalized with *N*-acetylcysteine for the removal of heavy ions, such as zinc (Zn^2+^), cadmium (Cd^2+^) and lead (Pb^2+^), in various media, including tap water, seawater and human serum. The results obtained demonstrated effectiveness in the removal of these metals in both environmental and biological samples, due to the high binding affinity of these ions to the *N*-acetylcysteine present in the cryogel.

Zhong et al.^52^ developed a cryogel based on poly­(vinylimidazole) and compared the adsorption processes of a poly­(imidazole) and poly­(vinylimidazole) cryogel for the removal of ions in aqueous solutions. It was observed that the poly­(vinylimidazole) cryogel presented a remarkable adsorption capacity for copper ions, reaching an efficiency of 99.99%, representing a 58-fold increase compared to poly­(imidazole).

Hou et al.^53^ in their research, investigated the removal of metal ions and oil in water using a poly­(vinylimidazole diacrylate-*co*-polyethylene glycol) cryogel. In this study, they achieved an efficiency of 97.5% in the removal of copper ions, indicating that the cryogel was able to remove oil from emulsion containing Cu­(II) in water. Thus, this material has potential to be used in wastewater cleaning processes.

## 5 Monolithic Affinity Columns for Lectin Isolation

Lectins are proteins of nonimmune origin that have carbohydrate-binding sites in their structure, and this binding occurs in a specific and reversible manner. Because of these binding sites, they can be used in the medical, biomedical, pharmaceutical and food industries. Lectins can be isolated from a variety of sources: plant and even microbial, and a method widely used in this isolation is purification by liquid chromatography using chromatographic columns.^54–56^

A chromatography column is a fixed bed used in liquid chromatography. This method is based on the identification and separation of substances through the physical and chemical interaction between the stationary phase and the mobile phase. The stationary phase is made up of solid particles packed into a column, while the mobile phase is a liquid solvent that percolates through the column.^57,58^

The column is a porous material which, due to its physical, morphological and hydrodynamic characteristics, allows compounds to be separated accurately and efficiently. Substances can bind to the bed of the stationary phase through physical or chemical interactions, depending on the material immobilized on the column. These interactions can include ionic bonds, hydrophobic interactions and affinity, which are the main chromatographic techniques when you want to isolate a substance with a high degree of purity.^2,7,59,60^

Lectins have carbohydrate-binding sites in their structures which, depending on their origin, are specific to one or more carbohydrates, known as carbohydrate recognition domains (CRDs).^4^ Due to the structure of lectins, the processes for isolating these molecules are based on chromatography columns that have the greatest possible interaction with the lectin of interest.

Most studies that seek to isolate lectins often use a gel filtration column (Sephadex G-75) combined with an ion exchange column (Diethylaminoethyl-Sepharose).^61^ Few studies use affinity columns immobilized with specific carbohydrates, such as chitin matrix,^7,62^ xanthan gum, or Sepharose-4B-Lactose.^63^

The commercial columns commonly used to purify lectins are generally more economically expensive than the macroporous monolithic columns currently being developed. Table 2 shows the types of commercial columns most frequently used to isolate lectins and their values, compared to those of cryogel columns.

**Table 2:** Commercial Columns for Lectin Purification Processes^a^

	commercial columns		
column type	value (real and dollar)	biomolecules under study	references
Amersham Biosciences Mono S PC 1.6/5 Precision Column	BRL 5.350, 41 (100 g)	emperor banana-BanLec	Wong and Ng^64^
Superdex 75 Increase 10/300 GL	BRL 14.457, 30 (100 g)		
	BRL 5.049, 27 (100 g)		
QAE-Sephadex A-50, (Pharmacia, Uppsala, Sweden)	BRL 3.980, 00 (100 g)	BanLec	Gavrovic-Jankulovic et al.^65^
	BRL 5.664, 00 (100 g)		
Mannose-Agarose	BRL 32, 180.00 (100 mL)	banana (BL) and garlic (GL)	Hinge et al.^66^
Sephadex G-75	BRL 5, 510.00 (100 g)	banana-BanLec	Wearne et al.^67^
Sephadex G-75	BRL 5, 510.00 (100 g)	banana-BanLec	De Camargo et al.^68^
DEAE Sephadex	BRL 5, 944.00 (100 g)		
Q-Sepharose (GE Healthcare, Hong Kong)	BRL 2, 910.00 (75 mL)	banana-BanLec	Chan and Ng^69^
Mono Q column (1 mL) (GE Healthcare, Hong Kong)	BRL 29.763, 36 (100 mL)		
Superdex 75	BRL 4.457, 30		
DEAE-cellulose	BRL 896, 72 (100 g)	Cicer arietinum-CAL	Gautam et al.^70^
SP Sephadex C-25	BRL 4, 618.00 (100 g)		
monolithic columns in development			
poly(AAm)	BRL 1, 031.00 100g	jackfruit-jacalin	Nascimento et al.^2^
poly(HEMA-GMAIL)	BRL 1, 337.00 (100 g)	lysozyme	Bayramoglu and Yakup Arica^17^
poly(lauryl methacrylate-divinylbenzene)	BRL 2, 913.00 (100 g)	-	Chen et al., 2016
2-hydroxyethyl methacrylate (HEMA)	BRL 781.00 (100 g)	human Immunoglobulin M	Bakhshpour et al.^71^

a Source: Author.

These commercial columns are generally more expensive when compared to monolithic columns, such as cryogels, which vary in price depending on the polymeric material used. Table 2 shows that columns produced from polyacrylamide form a column with physical, morphological and hydrodynamic characteristics that are essential for chromatographic processes for the purification of biomolecules. Also according to Table 2, there are few studies in development for the use of these columns for the isolation of lectins, when compared to the use of this same matrix for the purification of other biological compounds.^2,15,17,25,71^

Table 2 addresses the main columns used in lectin isolation. Each column used has a mechanism that allows for a more specific interaction with the biomolecule of interest. Here we will provide a summary of how each column interacts with lectins so that they bind to the column, improving their isolation.

When working with lectin, the main form of chemical interaction involved between a chromatographic column and the biomolecule is affinity, since lectin has a carbohydrate binding site in its structure, which favors the interaction of this molecule with chemical compounds present in the structure of chromatographic columns. Therefore, the first choice of which type of column to use for lectin isolation is between conventional columns or the more modern ones, such as cryogels, which are used to isolate a lectin, bearing in mind that these columns have a carbohydrate in their structure that is capable of interacting with the lectin under study, promoting greater interaction when compared to other types of chemical interaction, such as ion exchange interaction. Some columns used in this case are QAE-Sephadex A-5065, d-glucosamine.^72,73^

In the case of ion exchange, the interaction mechanism is based on electrostatic interaction, i.e., this interaction occurs between the surface charges of the lectin and the functional groups of the matrix, which may include diethylaminoethyl (DEAE), carboxymethyl (CM), quaternary ammonium, sulphopropyl, among others. An example of a commercial column used for the isolation of proteins and lectins is the SP Sephadex C-25 is a strong cation-exchange resin in which lectins interact through reversible electrostatic attractions between positively charged amino acid residues on the protein surface and negatively charged sulfonate groups immobilized on the dextran matrix. Binding occurs when the pH of the mobile phase is below the lectin’s isoelectric point, and elution is typically achieved by increasing ionic strength or adjusting pH, without involvement of carbohydrate-specific recognition.^70^

Thus, a range of columns is used for lectin isolation, either conventional commercial columns or cryogel columns, which are a newer type of chromatographic column. The focus of this study is precisely the use of macroporous monolithic columns, cryogel, as the object of study due to two major advantages when compared to usual commercial columns: lower flow resistance, greater efficiency in viscous samples and plant extracts, pressure drop, high tolerance of raw extracts, the bond with the ligand occurs directly through the bond on the surface of the cryogel and, above all, its morphological structure, which is predominantly a porous structure with interconnected pores.^2,4,6,7,17^

Gonçalves et al.^6^ conducted a study involving the synthesis of a cryogel based on acrylamide, bis-acrylamide, AGE, TEMED and APS, as ligands immobilized some carbohydrates such as *N*-acetyl-d glucosamine (d-GlcNAc), *N*-acetyl-d-galactosamine (d-GalNAc) and *N*-acetyl-d-mannosamine (d-ManNAc), which have an affinity for the lectin under study, in this case concanavalin A (ConA), obtained from the leguminous *C. ensiformis*. The results showed an adsorption capacity of 44.49 mg/g using ConA extracts for cryogels immobilized with d-GlcNAc.

In the study by Nascimento et al.^2^ a macroporous monolithic column with anionic and cationic properties was developed to purify Jacalina lectin from jackfruit seeds. The polymer matrix was based on acrylamide, bis-acrylamide, AGE, TEMED and APS. The ligands used for cation exchange were 2-acrylamido-2-methyl-1-propanesulfonic acid (AMPSA) and acrylic acid (ACRAC), and two for anion exchange, 2-(dimethylamino)­ethyl methacrylate (DMAEMA) and [2-(acryloxy)­ethyl] trimethylammonium chloride (DMAEA-Q). As a result, the purification process achieved a 61.87% lectin recovery yield when using the DMAEMA-functionalized column.

In the study conducted by Ferreira da Silva et al.,^13^ a cryogel synthesized with acrylamide, bis-acrylamide, AGE, TEMED and APS was developed. In this case, *N*-acetyl-D glycosamine (D-GlcNAc) was immobilized in the cryogel to purify a lectin from barley extract that has a lectin related to wheat germ agglutinin (WGA). This made it possible to observe the selectivity of the column for the separation of lectin in the extract, since there was a 40% reduction in the hemeagglutinating activity of the extract with a reduction of about 10% in protein.

In conclusion, lectins stand out as biomolecules of great interest due to their specific ability to interact with carbohydrates, which makes them highly functional in various areas, such as medicine, biomedicine, pharmaceuticals and the food industry. The isolation of these proteins, although widely carried out using classic chromatographic techniques - such as gel filtration and ion exchange columns—still faces challenges related to efficiency, specificity and cost. Therefore, further studies should be conducted to develop new monolithic columns focused on isolating lectins from different biological sources, since commercial columns have a higher economic value, thus making the isolation of these lectins more expensive.

## 6 Future Prospects

Cryogels offer a versatile and efficient platform for protein capture, combining high adsorption capacity, selectivity and reusability. Their structural and chemical properties allow applications in biotechnology and pharmaceutical industries, standing out as a promising alternative to conventional protein purification methods.^2,13,69^

Technological advances involving monolithic columns are focused on improving functionalization techniques, mainly using aminated carbohydrates and ion exchangers, which provide a one-step purification process, thus increasing the potential for simplifying and accelerating the lectin isolation process.^2,13^

Cryogels have emerged as a promising technology for the purification of lectins due to their macroporous structure and ability to immobilize specific ligands.^2,4,13^ Recent advances in cryogels for lectin purification include the effective immobilization of carbohydrates^4^ and the development of macroporous monolithic structures. These developments highlight the versatility and efficiency of cryogels in lectin purification, offering fast and economical solutions for biotechnological applications.^7^

Many advances have been made in the use of cryogels in emerging technologies such as bioengineering in bioseparation and cell therapy applications.^74^ Due to their characteristics, especially their porosity, cryogels have been used in syringe injection processes and in minimally invasive therapies. Applications in the incorporation of bioactive compounds have the potential to heal wounds and prevent infections.^11^

In summary, cryogels represent an innovative and multifunctional tool with great potential for applications in protein purification, especially lectins, as well as in emerging areas of bioengineering. Their macroporous structure, high selectivity and ability to immobilize specific ligands allow for faster, more economical and efficient processes, consolidating them as a promising alternative to conventional methods.

In addition, advances in functionalization techniques and in the construction of monolithic columns further expand their applications, both in the biotechnology industry and in cell therapies and minimally invasive strategies, highlighting the role of cryogels as a prominent platform at the interface between science and technological innovation.

## 7 Conclusion

This review allows us to emphasize the great advances in the development and application of cryogels, materials that have a wide range of applications in various areas, such as food, biology, medicine, biomedicine, pharmaceuticals and the environment. It is a product that is easy to synthesize and reproduce.

Much research is still needed into the development of supermacroporous monolithic columns, especially for application in the food industry. Thus, future research can be carried out with emphasis on this area, since many advances have been made in the medical/biomedical and pharmaceutical fields in the use of cryogels.
